# Evaluation of the effect of methamphetamine on traumatic injury complications and outcomes

**DOI:** 10.1186/s13722-018-0112-6

**Published:** 2018-03-29

**Authors:** Michael M. Neeki, Fanglong Dong, Lidia Liang, Jake Toy, Braeden Carrico, Nina Jabourian, Arnold Sin, Farabi Hussain, Sharon Brown, Keyvan Safdari, Rodney Borger, David Wong

**Affiliations:** 1 Department of Emergency Medicine, Arrowhead Regional Medical Center, Medical Office Building, Suite 7, 400 N Pepper Ave, Colton, CA 92324 USA; 2 Western University of Health Sciences, College of Osteopathic Medicine of the Pacific, 309 E 2nd St., Pomona, CA 91766 USA; 3 Department of General Surgery, Arrowhead Regional Medical Center, 400 N Pepper Ave, Colton, CA 92324 USA; 4 Department of Anesthesiology, Arrowhead Regional Medical Center, 400 N Pepper Ave, Colton, CA 92324 USA; 5 California University of Sciences and Medicine, 1405 W Valley Boulevard, Suite 101, Colton, CA 92321 USA

**Keywords:** Methamphetamine, Trauma, Length of stay, Hospital mortality, Traumatic outcome

## Abstract

**Background:**

This study investigates the impact of methamphetamine use on trauma patient outcomes.

**Methods:**

This retrospective study analyzed patients between 18 and 55 years old presenting to a single trauma center in San Bernardino County, CA who sustained traumatic injury during the 10-year study period (January 1st, 2005 to December 31st, 2015). Routine serum ethanol levels and urine drug screens (UDS) were completed on all trauma patients. Exclusion criteria included patients with an elevated serum ethanol level (> 0 mg/dL). Those who screened positive on UDS for only methamphetamine and negative for cocaine and cannabis (MA(+)) were compared to those with a triple negative UDS for methamphetamine, cocaine, and cannabis (MA(−)). The primary outcome studied was the impact of a methamphetamine positive drug screen on hospital mortality. Secondary outcomes included length of stay (LOS), heart rate, systolic and diastolic blood pressure (SBP and DBP, respectively), and total amount of blood products utilized during hospitalization. To analyze the effect of methamphetamine, age, gender, injury severity score, and mechanism of injury (blunt vs. penetrating) were matched between MA(−) and MA(+) through a propensity matching algorithm.

**Results:**

After exclusion, 2538 patients were included in the final analysis; 449 were patients in the MA(+) group and 2089 patients in the MA(−) group. A selection of 449 MA(−) patients were matched with the MA(+) group based on age, gender, injury severity score, and mechanism of injury. This led to a final sample size of 898 patients with 449 patients in each group. No statistically significant change was observed in hospital mortality. Notably, a methamphetamine positive drug screen was associated with a longer LOS (median of 4 vs. 3 days in MA(+) and MA(−), respectively, *p* < 0.0001), an increased heart rate at the scene (103 vs. 94 bpm for MA(+) and MA(−), respectively, *p* = 0.0016), and an increased heart rate upon arrival to the trauma center (100 vs. 94 bpm for MA(+) and MA(−), respectively, *p* < 0.0001). Moreover, the MA(+) group had decreased SBP at the scene compared to the MA(−) group (127 vs. 132 bpm for MA(+) and MA(−), respectively, *p* = 0.0149), but SBP was no longer statistically different when patients arrived at the trauma center (*p* = 0.3823). There was no significant difference in DBP or in blood products used.

**Conclusion:**

Methamphetamine positive drug screens in trauma patients were not associated with an increase in hospital mortality; however, a methamphetamine positive drug screen was associated with a longer LOS and an increased heart rate.

## Background

Methamphetamine is a potent stimulant that affects the central nervous system. Use of methamphetamine results in immediate effects that often include euphoria, aggression, erratic behavior, increased libido, emotional lability, and psychosis lasting on average for 6 to 12 h [1–4]. In 2015, an increasing trend of methamphetamine use in the United States among individuals 12 years and older was noted with an estimated 5.4% of the population having tried methamphetamine in their lifetime [5]. Geographically, methamphetamine use is most predominant on the West Coast and in the Midwest; however, the prevalence of use is rapidly spreading east across the United States [1, 5, 6]. This increase in methamphetamine use has been reflected in emergency departments (ED) around the country [1, 7–10]. The economic impact has also been significant [9, 11]. In 2005, the economic burden of methamphetamine use in the United States was estimated to be $23.4 billion [11].

There are extensive published reports on the deleterious cardiovascular and neurological effects of methamphetamine; however, there is a paucity of evidence pertaining to the impact of methamphetamine in the context of traumatic injury [6, 12–16]. Previous studies have demonstrated an association between methamphetamine use and an increased risk of traumatic injury [10, 17]. Yet studies assessing the rate of ambulance transport, percentage of hospital admissions, length of stay (LOS), and need for emergent surgery amongst this demographic are varied and infrequent [1, 6, 10, 17–19]. Additionally, positive methamphetamine screens amongst trauma patients in the ED have been associated with inconsistent findings concerning the impact of methamphetamine on mortality outcomes in a limited number of studies [17–19]. Though trauma patients often receive urine drug screening for methamphetamine upon admission at many centers, the value of this laboratory marker is poorly understood.

We seek to assess the impact of methamphetamine use in the context of traumatic injury and the impact of methamphetamine use on hospital mortality outcomes, LOS, and acute physiologic profile. A greater understanding of these variables may aid in improving clinical management and allocation of hospital resources.

## Methods

This retrospective chart review was undertaken at Arrowhead Regional Medical Center (ARMC). ARMC is a 456-bed acute care teaching facility and the only American College of Surgeons certified level II trauma center located in San Bernardino County, CA with over 92,000 visits annually. San Bernardino County is the largest county by area in the contiguous United States with a population of over two million.

Data were gathered from the trauma registry at ARMC. Trauma patients between 18 and 55 years old who were admitted between January 1st, 2005 and December 31st, 2015 were assessed for inclusion. During the study period, all trauma patients seen at our center underwent routine urine drug screening (UDS) and measurement of a serum ethanol level in the ED prior to admission. Exclusion criteria included patients with an elevated serum ethanol level (greater than  0 mg/dL) and UDS positive for any drug other than methamphetamine (including cocaine and cannabis). For trauma patients presenting to the trauma center with multiple visits, only the most recent visit was included in the analysis.

Patients were divided into two groups based on the presence of methamphetamine on urine drug screen (MA(+) vs. MA(−)). The MA(+) group included patients with a positive UDS for methamphetamine, negative UDS for cocaine and cannabis, and serum ethanol level less than 0 mg/dL. The MA(−) group was defined as patients with a triple negative UDS for methamphetamine, cocaine, and cannabis, and serum ethanol level less than 0 mg/dL. Routine UDS and serum ethanol level were performed using the Cobas 6000 analyzer series (Roche Diagnostics USA, Indianapolis, Indiana, USA). Though urine opiate levels were included in the UDS at our center, this data was not included in the study. The threshold to detect urine amphetamine concentrations was set at greater than 1000 ng/ml. Additional patient data collected included age, gender, injury severity score (ISS), mechanism of injury (blunt vs. penetrating), transfusion need during hospital stay, LOS, hospital mortality, and heart rate (normal adult resting heart rate is 60–100) and blood pressure (normal adult resting diastolic blood pressure [DBP] is less than 80 mmhg and systolic blood pressure [SBP] less than 120 mmhg) taken by first responders after arrival at the scene and by ED staff upon arrival to the trauma center. The ISS is derived from a validated scoring system used to describe overall injury severity based on the anatomic regions involved [20]. Major trauma (polytrauma) has been defined as ISS  greater than 15 and a higher ISS has been correlated with an increase in morbidity and mortality [21].

The primary outcome was mortality during hospital stay. Other outcomes included hospital LOS, total amount of blood products utilized during the hospital stay, heart rate taken at the scene and upon arrival to the trauma center, and SBP and DBP at the scene and upon arrival to the trauma center.

All statistical analyses were conducted using the SAS software for Windows version 9.3 (SAS Institute, Cary, North Carolina, USA). Descriptive statistics were presented as means and standard deviations for continuous variables, and frequencies and proportions for categorical variables. Chi square crosstab analysis was conducted to identify whether the proportion of penetrating trauma was comparable between the MA(+) and MA(−). If the proportions of penetrating trauma were statistically different between the MA(+) and MA(−) group, a propensity score 1–1 matching was conducted to select the same number of participants from the MA(−) to match the participants from the MA(+) group based on age, gender, ISS, and mechanism of injury (blunt vs. penetrating) using the package “MatchIt” in R. The choice of these four matching variables for the matching process was to eliminate the confounding effect on primary and secondary outcomes. After data were matched, comparison of continuous variables was conducted using the independent *T* test between the MA(+) and MA(−) groups. Comparison of categorical variables was conducted using the Chi square test. All statistical analyses were two-sided. *p* values < 0.05 were considered to be statistically significant. This study was approved by the Institutional Review Board at ARMC.

## Results

**Fig. 1:**
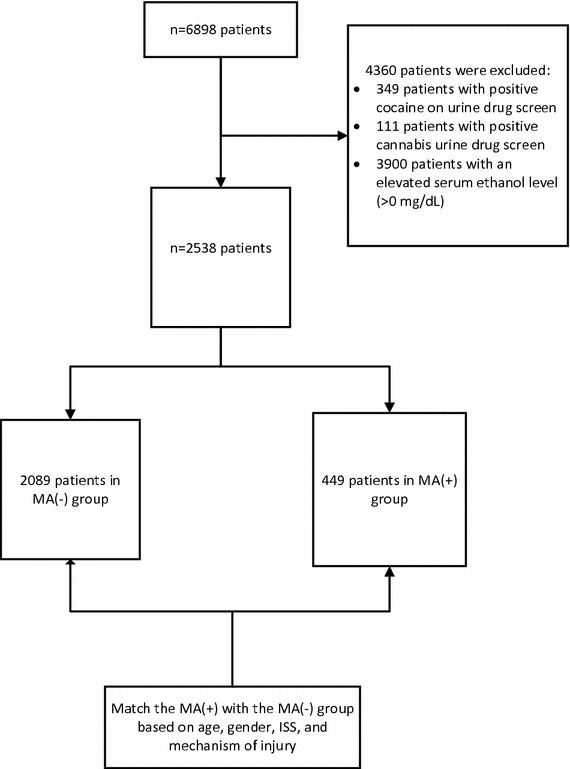
Patient selection flow chart. *MA(−) = a triple negative drug screen for methamphetamine, cocaine, and cannabis, and serum ethanol level < 0 mg/dL matched to the MA(+) group via propensity score matching. MA(−) was selected to match with MA(+) based on age, gender, ISS, and mechanism of injury. **MA(+) = a positive drug screen for methamphetamine, and negative for cocaine or cannabis, and serum ethanol level < 0 mg/d. ***ISS = injury severity score

Among the 6898 patients included in the original database, 3900 patients were excluded due to elevated serum ethanol levels, 349 patients were excluded due to positive cocaine on UDS, and 111 patients were excluded due to positive cannabis on UDS, which led to a cohort of 2538 patients. A total of 449 patients were positive for MA(+) and 2089 patients were MA(−) (see Fig. 1). A selection of 449 patients in the MA(−) group was matched with MA(+) group based on age, gender, ISS, and mechanism of injury. This led to a final sample size of 898 patients with 449 patients in each group.

No difference in hospital mortality was noted between the two groups (3.3 vs. 2.7% for MA(+) and MA(−), respectively, *p* = 0.5577). Patients in the MA(+) group, however, had a longer hospital LOS (a median of 4 vs. 3 days for MA(+) and MA(−), respectively, *p* = 0.0001). MA(+) patients also utilized less blood products (2054 ml vs. 2481 ml for MA(+) and MA(−), respectively, *p* = 0.3547), though blood product utilization was not statistically different.

A comparison between the MA(+) and MA(−) groups was conducted to assess their acute physiologic profile (Table 1). Patients in the MA(+) group had a statistically significant increase in pulse taken both on scene (103 vs. 94 beats per minute for MA(+) and MA(−), respectively, *p* < 0.0001) and upon arrival to the trauma center (100 vs. 94 beats per minute for MA(+) and MA(−), respectively, *p* = 0.0063). Moreover, patients in the MA(+) group had a statistically significant decrease in SBP at the scene than patients in the MA(−) group (127 vs. 132 beats per minute for MA(+) and MA(−), respectively, *p* = 0.0149), however, SBP was no longer statistically different when patients arrived at the trauma center (*p* = 0.3823). There was no difference on DBP regardless whether at the scene or upon arrival to the trauma center (both *p* values > 0.05).

**Table 1:** Comparison between positive (MA(+)) and negative (MA(−)) methamphetamine drug screens

	MA(+) n = 449	MA(−) n = 449	*p* value
Age	34.18 ± 10.75	34.23 ± 11.1	0.9367
Gender			0.6736
Female	90 (20%)	85 (18.9%)	
Male	359 (80%)	364 (81.1%)	
Mechanism of Injury			0.6116
Blunt	309 (68.8%)	316 (70.4%)	
Penetrating	140 (31.2%)	133 (29.6%)	
Injury severity score	14.76 ± 10.86	14.72 ± 10.7	0.9548
Hospital Mortality			0.5577
Alive	434 (96.7%)	437 (97.3%)	
Dead	15 (3.3%)	12 (2.7%)	
Blood product			0.2452
No blood product received (n)	351 (78.2%)	365 (81.3%)	
Any Blood product received (n)	98 (21.8%)	84 (18.7%)	
Units of Total amount of blood product received (mL)	2053.65 ± 2824.25	2480.64 ± 3383.46	0.3547
Median Hospital LOS in days (Q1, Q3)	4 (2, 8)	3 (1, 7)	0.0001
Pulse on scene	102.6 ± 22.06	94.31 ± 20.97	< 0.0001
SBP on scene	126.89 ± 26.9	131.52 ± 24.34	0.0149
DBP on scene	83.31 ± 18.29	85.23 ± 19.73	0.2537
Pulse on arrival to the trauma center	99.96 ± 22.76	94 ± 20.61	< 0.0001
SBP on arrival to the trauma center	139.78 ± 25.92	141.25 ± 24.46	0.3823
DBP on arrival to the trauma center	86.59 ± 20.19	87.03 ± 19.48	0.7449

* MA(−) = a triple negative urine drug screen for methamphetamine, cocaine, and cannabis as well as serum ethanol level < 0 mg/dL matched to the MA(+) group via propensity score matching. MA(+) = a positive urine drug screen for methamphetamine, and negative for cocaine or cannabis as well as serum ethanol level < 0 mg/dL

** All continuous values were presented as mean ± SD or median (Q1, Q3) for hospital LOS. Categorical variables were presented as frequency with column percentages inside the parenthesis

*** *LOS* length of stay,* SBP* systolic blood pressure,* DBP* diastolic blood pressure

## Discussion

Although it has been widely established that methamphetamine use results in toxic effects on the body and increases the likelihood of sustaining a traumatic injury, the effects of methamphetamine on traumatic injury outcomes in the post-injury period remain unclear. The current study suggests no change in hospital mortality outcomes and a longer hospital LOS in trauma patients with positive methamphetamine drug screens. With respect to the association between a positive methamphetamine drug screen and trauma patient mortality, Yegiyants et al. [18] demonstrated a conflicting trend toward reduced mortality among trauma patients who had a positive methamphetamine drug screen. However, Hadjizacharia et al. [19] noted no significant correlation. Taken together, it appears that a positive methamphetamine drug screen does not correlate with trauma patient mortality. With regards to hospital LOS, the findings of the current study are consistent with select prior reports suggesting that minimally injured trauma patients with positive methamphetamine drug screens have a significantly longer hospital LOS [6, 17]. Yet other studies have reported that trauma patients with positive methamphetamine drug screens did not have an increased LOS in the intensive care unit (ICU) or the hospital, but may be more likely to be admitted to the ICU [17, 19].

We further assessed the acute physiologic profile of the trauma patients studied. Given the known impact of methamphetamine on an individual’s physiologic status (i.e. tachycardia, vasoconstriction, vasospasm) [22], we hypothesized that tachycardia and an elevated blood pressure among patients with methamphetamine positive drug screens may offer an explanation to the observed mortality outcomes noted in the aforementioned studies [18, 19]. An unstable acute physiologic profile may have elicited a higher level of care from clinicians providing a possible explanation for the reduced and unchanged mortality outcomes previously observed in patients with positive methamphetamine screens. Though a significant difference in pulse rate was found amongst trauma patients with positive methamphetamine screens in this study, this difference was minimal and likely clinically insignificant. A significant difference in blood pressure was also noted at the scene amongst trauma patients with positive methamphetamine drug screens; however, this difference was also minimal and likely clinically insignificant. Of note, given that methamphetamine metabolites may be present on UDS for approximately two to five days after last use without substantial lasting physiologic effects, it may be difficult to attribute all of the observed findings to methamphetamine. To our knowledge, no previous study has assessed the impact of positive methamphetamine drug screens on the acute physiological profile in trauma patients.

Reasons for the increased hospital LOS observed among trauma patients with methamphetamine positive drug screens in this study were likely multifactorial. One contributing factor may be a delay in surgical care due to a positive methamphetamine drug screen upon admission. Based on our hospital anesthesiology protocols, patients suffering from open fractures or other injuries requiring emergent procedures will undergo surgery regardless of drug screen results upon admission; however, patients who have positive methamphetamine drug screens and require non-emergent surgeries may wait up to five days for a subsequently negative methamphetamine drug screen on repeat toxicology testing. As a potent sympathomimetic, methamphetamine and its metabolites have been shown to change the minimum alveolar concentration of inhaled anesthetics as well as both increase and decrease the required dose of anesthetic agents [23–25]. By altering anesthetic requirements, amphetamines have been implicated in cases of cardiac arrest, severe intraoperative intracranial hypertension, and reflex hypotension during general anesthesia [26–29]. Nonetheless, the impact of anesthesiology protocols on LOS may be institution dependent.

An additional and widely generalizable contributory factor to the increased LOS observed may have been an unreliable patient exam secondary to acute methamphetamine intoxication. This may have contributed to an extended observation period and extra diagnostic studies, potentially leading to an increased LOS. Alternatively, a higher frequency of comorbidities seen in chronic methamphetamine users, complications associated with methamphetamine withdrawal, or issues concerning placement after discharge may have contributed to the observed difference in LOS [13]. Future studies are warranted to assess the impact of these factors on LOS among patients with positive methamphetamine screens.

Irrespective of the root cause, the potential downstream effects resulting from added hospital days are numerous. With an average inpatient hospital day costing over $7000 at our center, an additional hospital day for patients with a positive methamphetamine screen places significant financial burden on the patient, hospital, and healthcare system. Increased costs associated with the hospitalization of trauma patients with positive methamphetamine screens have been consistently demonstrated in previous literature [6, 9, 17]. Other consequences include the diversion of trauma care resources away from other patients and increased risk of hospital-acquired infections due to an extended LOS.

## Limitations

This study has several limitations. First, patients seen at our center suffering from traumatic injury during the study period underwent routine drug and alcohol screening prior to admission; however, those directly admitted or who require emergent surgery for life-threatening injuries may not have undergone drug screening. Though a small subset of patients with methamphetamine positive drugs screens may not have been included, the impact on our results was likely minimal given our matching algorthim.

Second, this study did not assess rates of ICU admission or ICU LOS. However, analysis of hospital mortality, injury severity, and transfusion need yielded no significant differences. Together, these findings suggest similar hospital courses for trauma patients with positive or negative methamphetamine screens in our study. The study also did not assess the need for surgical intervention amongst our patient sample. Combined, these two aspects significantly limit our understanding of the exact etiology leading to the observed increase in LOS.

Third, this study did not exclude patients with urine drug screens that were positive for opiates. Our sample included patients suffering from acute traumatic injuries, many of whom received analgesic drugs including opioid derived drugs in both the prehospital setting and upon arrival to the trauma center. Approximately 33% of patients in the MA(+) group screened positive for opiates on urine drug screen and 17% of patients in the MA(−) group screened positive for opiates. Unfortunately, we could not differentiate between opiate positive urine drugs screens that were due to analgesic cause versus illicit use. However, given the differing mechanism of action of methamphetamines and opioids, we feel that study outcomes were minimally affected.

A final limitation includes both the potential for false positive and false negative drug screens. Amphetamine and methamphetamine have been documented as the most commonly reported illicit drugs with a false-positive UDS due to their cross-reactivity with many commonly prescribed drugs like bupropion, trazodone, chlorpromazine, promethazine, ranitidine, and various antihistamines and decongestants [30]. Additionally, false negative results arise because toxicology screening for popular amphetamine analogues such as cathinone, a beta-ketone amphetamine moiety that has physiologic properties remarkably similar to that of methamphetamine, are underdeveloped due to the novelty of such analogues [31–33].

## Conclusion

The current study suggests that trauma patients with positive methamphetamine drug screens do not have a significant difference in hospital mortality outcomes when compared to those with negative methamphetamine drug screens. Despite these findings, routine urine toxicology screening and measurement of serum ethanol level amongst trauma patients may still be warranted as these findings could assist in medical decision making throughout a patient's hospital course and disposition. Future studies are warranted to further assess the factors that contribute to an increased LOS for trauma patients with positive methamphetamine screens and to develop a greater insight into the clinical value of urine drug testing for trauma patients upon admission.

## Abbreviations

ED: emergency department
LOS: length of stay
ARMC: Arrowhead Regional Medical Center
UDS: urine drug screening
MA(+): methamphetamine positive
MA(−): methamphetamine negative
ISS: injury severity score
SBP: systolic blood pressure
DBP: diastolic blood pressure
